# Genetic Characterization of Feline Leukemia Virus from Florida Panthers

**DOI:** 10.3201/eid1402.070981

**Published:** 2008-02

**Authors:** Meredith A. Brown, Mark W. Cunningham, Alfred L. Roca, Jennifer L. Troyer, Warren E. Johnson, Stephen J. O’Brien

**Affiliations:** * National Cancer Institute, Frederick, Maryland, USA; † Florida Fish and Wildlife Conservation Commission, Gainesville, Florida, USA; ‡ University of Illinois at Urbana-Champaign, Urbana, Illinois, USA; § SAIC-Frederick, Frederick, Maryland, USA

**Keywords:** Communicable diseases, emerging, leukemia virus, feline, molecular biology, immunodeficiency virus, research

## Abstract

From 2002 through 2005, an outbreak of feline leukemia virus (FeLV) occurred in Florida panthers (*Puma concolor coryi*). Clinical signs included lymphadenopathy, anemia, septicemia, and weight loss; 5 panthers died. Not associated with FeLV outcome were the genetic heritage of the panthers (pure Florida vs. Texas/Florida crosses) and co-infection with feline immunodeficiency virus. Genetic analysis of panther FeLV, designated FeLV-Pco, determined that the outbreak likely came from 1 cross-species transmission from a domestic cat. The FeLV-Pco virus was closely related to the domestic cat exogenous FeLV-A subgroup in lacking recombinant segments derived from endogenous FeLV. FeLV-Pco sequences were most similar to the well-characterized FeLV-945 strain, which is highly virulent and strongly pathogenic in domestic cats because of unique long terminal repeat and envelope sequences. These unique features may also account for the severity of the outbreak after cross-species transmission to the panther.

## Abstract 2

The emergent strain of FeLV, a novel subgroup A, was probably transmitted to panthers by a domestic cat.

The Florida panther (*Puma concolor coryi*) is the only remaining puma (also called cougar or mountain lion) population east of the Mississippi River in North America. This population, which is confined to a small portion of southern Florida, was originally described as 1 of 30 subspecies of puma (*1*). By the 1970s, Florida panther numbers diminished to ≈30 because of hunting and habitat destruction. Since the early 1980s, the population has been studied extensively by monitoring a large proportion of adults by radio telemetry (*2*–*5*). In the early 1990s, concern over the fate of the population increased as signs of inbreeding and loss of genetic diversity were reported. These observations included low levels of genetic variation, high levels of sperm abnormalities, and increased incidence of heart defects relative to other puma populations and felids in general (*2*,*3*). In 1995, faced with the compounding effects of reduced genetic variation, probable depression of numbers from inbreeding, and evidence of compromised health, wildlife managers released 8 female Texas pumas into southern Florida to increase genetic variation and ameliorate the physiologic effects of inbreeding. Subsequently, increases were noted in the population of individuals of mixed genetic heritage, genetic variation, and population size; a decrease was noted in incidence of deleterious physiologic traits in crosses between the pure Florida panthers and the Texas females (*4*).

The Florida panther population, as well as other North and South American puma populations, has historically tested negative for exposure to or infection by feline leukemia viruses (FeLVs). A serosurvey of 38 free-ranging Florida panthers sampled during 1978–1991 reported complete absence of FeLV antigen (*3*). However, since early 2001, 23 panthers (>33% of the population) were found to be positive for FeLV antibodies, and at least 5 adult panthers were positive for FeLV antigen and subsequently died. In the 3 panthers available for necropsy, evidence was found of diseases compatible with FeLV infection (*5*). We describe the molecular genetic characterization of circulating FeLV strains isolated from the 2001–2005 outbreak and compare them with FeLV strains isolated from domestic cats.

FeLV is transmitted horizontally among domestic cats through body secretions (*6*) and was the first retrovirus shown to cause both neoplastic and degenerative disorders (*7*,*8*). Like other retroviruses, FeLV induces immunosuppression in its host. Although the mechanism of immunopathogenesis is unclear, viral envelope proteins may be involved (*9*). FeLV envelope (*env*) and the long terminal repeat (LTR) sequences have been suggested as being involved in determination of disease sequelae, virus transactivation, and virus replication (*10*–*12*). There are 4 naturally occurring viral subgroups of exogenous FeLV (A, B, C, and T) that are distinguished genetically by sequence differences in the *env* gene and functionally by receptor interactions required for cell entry (*13*). FeLV-A is the predominant subgroup circulating in feral cats and is often only weakly pathogenic (*14*). FeLV-B, -C, and -T subgroups arise in vivo through recombination between exogenous FeLV strains and domestic cat endogenous FeLVs (*8,**15*). The endogenous feline leukemia provirus sequences are present in the genome of the domestic cat and are transmitted vertically as integral components of the germline (*16*). Endogenous feline leukemia virus sequences by themselves do not produce infectious virus. However, the pathogenic subgroups, FeLV-B, -C, and –T, are generated by recombination in the *env* region between exogenous subgroup A virus and endogenous proviral sequences (*8*). FeLV-A, -B, -C, and -T are often associated, respectively, with thymic lymphoma of T-cell origin (*17*), tumor formation (*18*), aplastic anemia and bone marrow dysfunction (*17*), and lymphoid depletion and immunodeficiency disease (*13*). We used viral genome sequence and phylogenetic analyses to identify and characterize the virulent and pathogenic FeLV in Florida panthers and compare it with FeLV strains in the domestic cat.

## Materials and Methods

### Sample Collection and Testing

**Figure 1:**
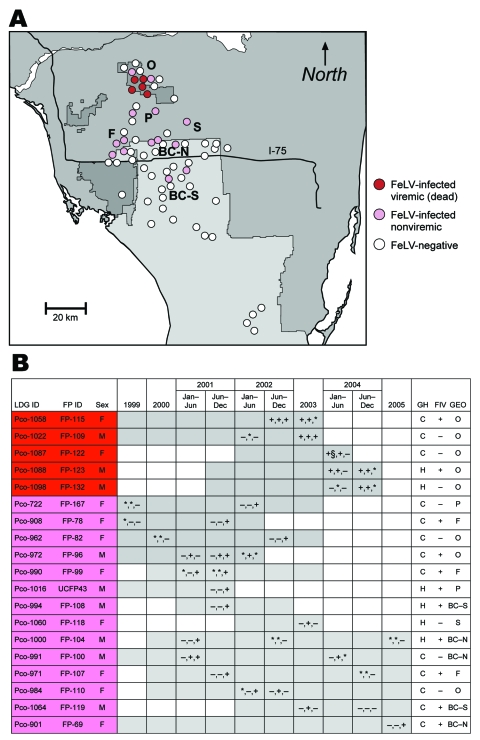
A) Prevalence and distribution of 19 Florida panthers, sampled 1999–2005, showing evidence of feline leukemia virus (FeLV) exposure. All antigen-positive panthers (red) are clustered in the Okaloacoochee Slough State Forest (O). PCR-positive and/or antibody-positive (pink) pumas were found there also, as well as in the surrounding areas including Florida Panther National Wildlife Refuge (F), private lands (P), Big Cypress Seminole Indian Reservation (S), and Big Cypress North and South (BC-N, BC-S respectively). All but 2 infected panthers were found north of Interstate 75. B) Information on affected panthers. Gray shading indicates timeline for monitoring of individual panthers until death. Symbols within gray boxes indicate presence (+), absence (–), or no data (*) for FeLV antigen in serum, FeLV sequence recovered by PCR, or presence of antibodies against FeLV in serum, respectively. FP-122 was antigen negative when tested 1 month previously (§). LGD ID, Laboratory of Genomic Diversity identification number; FP ID, Florida panther identification number; GH, genetic heritage; FIV, feline immunodeficiency virus; GEO, geographic locale; C, canonical (pure) Florida panther; H, Texas hybrid.

Blood and tissue samples were collected from 61 free-ranging pumas captured during 1988–2004, mainly from south Florida. Samples were stored at –70°C and tested for FeLV antigen and antibody and for feline immunodeficiency virus (FIV) by Western blot as described (*5*) (Figure 1).

### PCR Amplification of Proviral DNA

**Figure 2:**
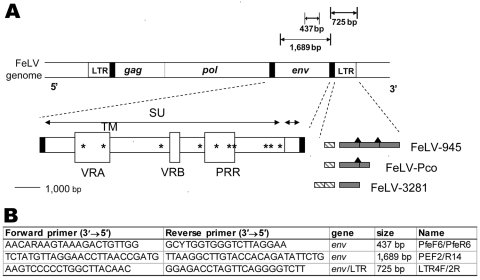
A) Diagram of the feline leukemia virus (FeLV) genome showing the PCR products obtained from FeLV-Pco *env* and long terminal repeat (LTR) genes. Envelope gene surface (SU) and transmembrane (TM) subunits, variable regions A and B (VRA and VRB) and the proline-rich region (PRR), 3’ LTR enhancer element(s) (hatched rectangle), signature 21-bp repeat(s) (gray shading), and putative c-Myb binding sites (black triangles) (*12*) are depicted for FeLV-945, FeLV-Pco, and FeLV-3281A . Unique signature amino acid residues found only in FeLV-945 and FeLV-Pco are marked by asterisks (see Figure 5). B) Primer pair PfeF6/PfeR6 was designed to detect all FeLV subgroups.

Genomic DNA was isolated from leukocytes, lymph nodes, spleen, intestines, or bone marrow of 61 panthers, including all that were positive for FeLV antigen and antibody. Proteinase K digestion was followed by standard extraction using the QIAGEN DNeasy tissue DNA extraction kit (#69504; QIAGEN, Valencia, CA, USA). Isolated DNA was visualized by electrophoresis on a 1% agarose gel and quantified by using a UV spectrophotometer (Bio-Rad, Hercules, CA, USA). PCR primers were designed from the conserved regions of *env* and LTR sequences of domestic cat FeLV (GenBank accession nos. M18247, M18248, M12500, AY374189, X00188, M14331, M23025, AY364318). PCR primers amplifying *env* (437 bp and 1,700 bp) and *env*/LTR (725 bp) are listed in Figure 2. The forward *env*/LTR primer (LTR4) was designed by using panther FeLV (FeLV-Pco) envelope sequence additionally.

PCR was performed by using ≈50 ng of genomic DNA in a 50-μL reaction with 50 mmol/L KCl; 10 mmol/L Tris-HCl (pH 8.3); 1.mmol/L MgCl_2;_ 0.25 mmol/L each of dATP, dCTP, dGTP, and dTTP; 2 mmol/L of each primer; and 2.5 U of Taq Gold polymerase (Applied Biosystems, Foster City, CA, USA). PCR was run on a GeneAmp PCR system 9700 thermocycler (Applied Biosytems) under the following conditions: 9 min 45 s at 95°C; then a touchdown of annealing temperatures to reduce nonspecific amplication, always starting with 20 s at 94°C; then 30 s at 60°C (3 cycles), 58°C (5 cycles), 56°C (5 cycles), 54°C (5 cycles), 52°C (5 cycles), or 50°C (22 cycles), and then 30 s (437-bp *env)*, 1 min (LTR) or 2 min 20 s (1,698-bp *env*) at 72°C for extension; and a final extension at 72°C for 7 min. PCR products were examined after electrophoresis on a 1% agarose gel. Primers and unincorporated deoxynucleotide triphosphates were removed by using Microcon YM (Millipore, Billerica, MA, USA) technology or exonuclease I and shrimp alkaline phosphatase (Amersham, Piscataway, NJ, USA) (Figure 2). Representative PCR products from independent amplifications were cloned and sequenced. For the *env* and LTR sequences, products were cloned from 4 PCR products each (Figure 2). Cloning was performed with a TOPO-TA cloning kit (Invitrogen, Carlsbad, CA, USA) according to the manufacturer’s instructions. DNA was isolated from 6 to 16 clones from each reaction product by using a QIAGEN Miniprep Kit. Sequences were obtained from clones by using internal primers in standard ABI BigDye terminator (Applied Biosystems) reactions. Anticontamination measures were taken at all steps of PCR amplification and after PCR processing. Pre-PCR setup was performed in a laminar flow hood, DNA was added in a free-standing containment hood in a separate room, and all post-PCR manipulations were performed under a fume hood in a third room. All surfaces were washed with a 10% bleach solution, and each hood was exposed to UV light for 30 min before and after use. PCR tubes with individual lids, rather than 96-well plates, were used and kept closed except when reagents and DNA were being added or aliquots were extracted for use. DNA tubes were opened only under their designated hoods; to avoid cross-contamination, tubes were never open simultaneously. Water and a sample from an FeLV-negative puma were run with every reaction as negative controls. Positive controls of known sequence were also run for each reaction: 1 from a domestic cat, 1 from a known seropositive Florida panther (FP-115 or FP-122), or both.

### Phylogenetic Analysis

Sequences from *env* and LTR were analyzed separately. For analysis relative to known domestic cat FeLV sequences, we included FeLV-945, FeLVA-3281, FeLVA-61E, FeLVA-Glasgow-1, FeLVC-Sarma, FeLVB-Rickard, SM-FeSV, enFeLV-AGTT (accession nos. AY662447, M18248, M18247, M12500, M14331, X00188, M23025, AY364318 respectively) (*env*) and FCA-945, FCA-934, FeLVA-3281, and FeLVA-Glasgow-1 (accession nos. AY374189, AY374184, M18248, and M12500, respectively) (LTR). Nucleotide sequences were compiled and aligned for subsequent phylogenetic analysis by ClustalX (*19*) and verified visually (*20*). MODELTEST (*21*) was used for *env* and LTR analysis to estimate the optimal model of sequence evolution; these settings were incorporated into subsequent analyses. Minimum-evolution trees were constructed from models of substitution specified by MODELTEST; starting trees were obtained by the neighbor-joining method, followed by application of a tree-bisection-reconnection branch-swapping algorithm during a heuristic search for the optimal tree. Maximum-parsimony analysis used a heuristic search of starting trees obtained by stepwise addition and followed by tree-bisection-reconnection. Maximum likelihood parameters specified by MODELTEST selected the general time-reversible model of substitution; they included empirical base frequencies and estimated rate matrix and corrected for among-site rate variation (γ distribution). A bootstrap analysis that used 1,000 iterations was performed with each method. Amino acid residue alignments were generated by using MacClade 3.05 (*20*) and ClustalX. Sequences were inspected for homoplasies. Nucleotide sequences were translated to protein, and genetic distances were calculated in MEGA 3.0 (*22*) by using the Tajima-Nei (nucleotide) and Dayhoff (amino acid) algorithms. The sequences of FeLV-Pco *env* and LTR were deposited in GenBank under accession nos. EU189489–EU189498.

## Results

### FeLV Serosurvey and PCR Amplification

The first sign of an emerging outbreak of FeLV in the free-ranging Florida panther population was the 2001 detection of FeLV antibodies, FeLV proviral PCR, or both, in 8 pumas from the Florida Panther National Wildlife Refuge, private lands, or the northern range of Big Cypress Swamp (Figure 1). Antigen-positive results and documented death compatible with FeLV infection first occurred in FP-115 in 2002 near the Okaloacoochee Slough State Forest (*5*). With the exception of FP-108 and FP-119, found in the central region of Big Cypress National Park, all 19 other FeLV-exposed panthers were found north of Interstate 75 (Figure 1) (*5*). During the next 2 years, 4 additional antigen-positive panthers died; FeLV-related disease was suspected for 2 (FP-123 and FP-132) and confirmed for 2 (FP-109 and FP-122) (*5*). Additionally, 8 panthers (FP-67, FP-78, FP-82, FP-96, FP-99, UCFP43, FP-108, FP-118) that were antigen negative but seropositive or PCR positive for FeLV died during the outbreak, but their deaths were not attributed to FeLV (*5*).

Retrospective screening of 6 panthers (FP-67, FP-78, FP-82, FP-109, FP-122, FP-132) for antibody or antigen or by PCR demonstrated that they had not had FeLV infection before this outbreak. FP-96 in the Florida Panther National Wildlife Refuge was one of the first to have documented FeLV exposure; this panther displayed a latent infection, being PCR positive in 2001 and in 2002. Three panthers (FP-104, FP-107, FP-119) likely cleared the virus; after initial positive test results, they were seronegative on follow-up testing. Positive FIV antibody results by Western blot were found for 11 of the 19 FeLV-exposed and 2 of the 5 clinically affected panthers (Figure 1). An analysis of 21 microsatellites (short tandem repeats) showed that 6 of the 19 FeLV-exposed and 2 of the 5 antigen-positive panthers were crosses with some Texas heritage and that the rest were pure Florida panthers (W.E. Johnson et al., unpub. data).

### Phylogenetic Analysis

**Figure 3:**
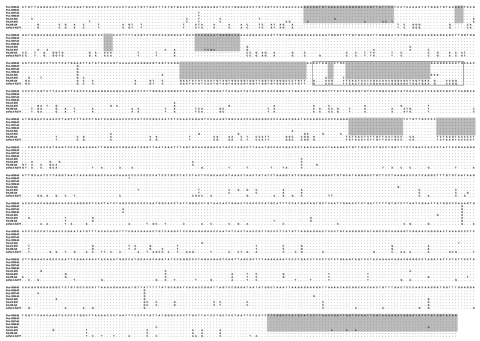
Excerpt of *env* nucleotide sequences. The shaded regions identify indels where FeLV-Pco sequence resembles that of feline leukemia virus A (FeLV-A), ruling out recombination with dissimilar endogenous FeLV sequences as represented in enFeLV-AGTT (bottom). Puma sequences, with year of sampling (for example FeLV-Pco-1058-03 was sampled in 2003); domestic cat subgroup A (FeLVA-945 and FeLVA-61E), recombinant (FeLVB-GA), and endogenous (enFeLV-AGTT) sequences are also shown. Matches to the reference sequence (Pco-1058-02) are indicated by a dot. Gaps are indicated by a dash. (Expanded Figure)

**Figure 4:**
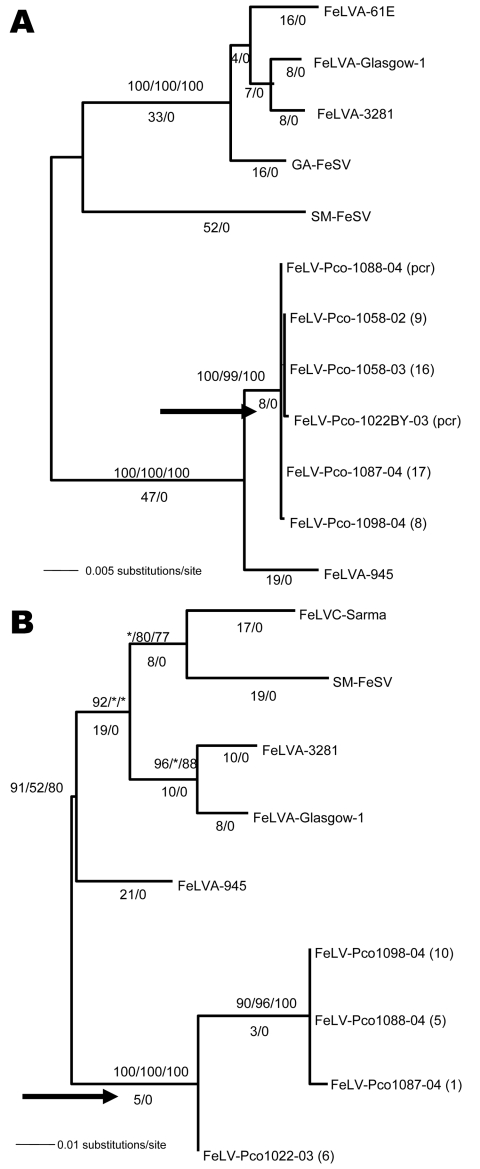
Phylogenetic trees of panther feline leukemia virus (FeLV-Pco) and domestic cat FeLV nucleotide sequences. A) Midpoint rooted maximum-likelihood phylogram based on 1,698 bp of *env* sequences. B) Midpoint rooted maximum-likelihood phylogram based on 463 bp of 3’ long terminal repeat (LTR) sequences. Consensus FeLV-Pco sequences of clones generated from 5 *env* and 4 LTR panthers and reference domestic cat sequences are shown. The number of FeLV-Pco–cloned PCR products used in each consensus sequence is indicated in parentheses. The arrow indicates the monophyletic clade of all FeLV-Pco sequences. A similar topology, including the monophyletic clade, was obtained by using the different FeLV-Pco clone sequences rather than a consensus. The year of panther sampling is indicated as a suffix, e.g., Pco-1088-04 was sampled in 2004. Where maximum-likelihood tree was congruent with maximum parsimony tree, branch lengths are indicated below branches. Number of homoplasies is indicated after the branch length. Bootstrap values are shown (maximum parsimony/minimum evolution/maximum likelihood). The score (–ln likelihood) of the best maximum-likelihood tree was *env* 3615.01706, LTRs 1836.05922 (best tree found by maximum parsimony: *env* length = 221, consistency index [CI] = 0.941, retention index [RI] = 0.963; LTR length = 132, CI = 0.871, RI = 0.787).

An alignment of FeLV-Pco, FeLV-A, FeLV-B, and endogenous *env* nucleotide sequence (Figure 3) established the concordance of FeLV-Pco with subgroup A and found a lack of recombination of FeLV-Pco with endogenous FeLV-Pco sequence. The absence of endogenous sequences was not unexpected because pumas and other cats outside of the genus *Felis* do not carry endogenous FeLV sequences (*23*,*24*). The FeLV-Pco was classified as subgroup A on the basis of this lack of evidence for recombination with endogenous FeLV across 1,794 bp of FeLV-Pco *env* sequence (Figure 3) and on the basis of in vitro receptor utilization studies (*5*). The aligned sequences of the LTRs and the *env* region were analyzed as separate datasets. For both datasets, phylogenetic analyses identified the FeLV-Pco sequences as monophyletic (Figure 4). Each had strong bootstrap support for a clade containing all FeLV-Pco but none of the previously sequenced domestic cat FeLVs (Figure 4). This pattern was consistent with a recent and focal introduction of the virus. Furthermore, the 376-bp nucleotide *env* sequence obtained from the earliest cases of FeLV exposure (Pco-972 and Pco-991, found respectively in the Florida Panther National Wildlife Reserve and northern Big Cypress National Preserve), were identical in sequence to the later FeLV cases found in the Okaloacoochee Slough State Forest (Figure 2; Appendix Figure 1). On the basis of >50 cloned envelope sequences (Figure 4; Table), the FeLV-Pco viruses associated with this outbreak were highly conserved. The mean percentage nucleotide and amino acid sequence differences of the FeLV *env* gene among FeLV-Pco sequences were 0.4% (nucleotide) and 0.1% (amino acid). Of published FeLV sequences available in GenBank, the closest strain was the domestic cat virus FeLV-945, according to LTR and *env* sequence comparisons (Figure 4); calculated differences were only 1.5% (nucleotide) and 3.5% (amino acid) between FeLV-Pco and FeLV-945 *env* sequences (Table).

**Table:** Mean percent amino acid and nucleotide *env* sequence differences of feline leukemia virus subgroups, FeLV-945, and FeLV-Pco strains* www.cdc.gov/EID/content/14/2/252-T.htm

Strain	FeLV A†	FeLV B‡	FeLV C§	FeLV-945¶	FeLV Pco#
FeLV A	**1.8**, 3.8	10.3	6.6	6.4	6.1
FeLV B	**19.1**	NA	13.2	14	13.3
FeLV C	**16.3**	**28.7**	**14.2**, 6.2	7.3	7.4
FeLV 945	**15.2**	**30.1**	**16.4**	NA	1.5
FeLV Pco	**14.3**	**28.2**	**16.7**	**3.5**	**0.4**, 0.1

*FeLV, feline leukemia virus. Shaded gray boxes contain mean percent sequence differences within strain. **Boldface** indicates mean percent amino acid *env* sequence differences, 1,689 bp (see Figure 2). Nucleotide differences are above diagonal. †M18247, M18248, M12500, AF052723. ‡X00188. §M14331, M23025 ¶AY374189. #Pco-1058-02 (9 cloned sequences), Pco-1058-03 (16 cloned sequences), Pco-1087-04 (17 cloned sequences), Pco-1098-04 (8 cloned sequences).

**Figure 5:**
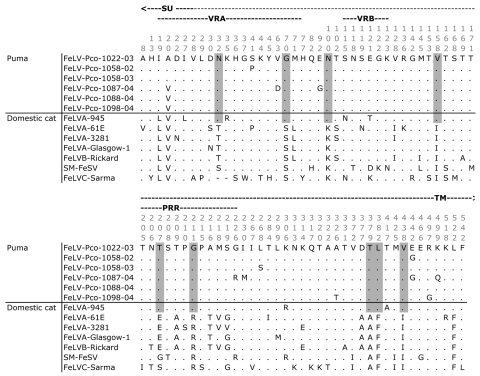
Variable sites in the amino acid alignment of panther feline leukemia virus (FeLV-Pco) and domestic cat FeLV *env* sequences (1,689 bp). Surface glycoprotein (SU), transmembrane (TM), variable region A and B (VRA and VRB), and proline-rich region (PRR) locations are indicated. Horizontal line separates sequences of puma (above) and domestic cat (below). The 10 amino acid residues in this region unique to FeLV-945 and FeLV-Pco sequences are shaded in gray. Matches to the reference sequence are indicated by dots; gaps are indicted by dashes.

Because FeLV-945 is well characterized and highly virulent in the domestic cat (*11*,*25*), sequence elements associated with disease determination (*env*) and transcription enhancement (LTR) in FeLV-945 were examined in FeLV-Pco. In the envelope protein, 10 signature amino acid residues (found within the surface glycoprotein) that were shared between FeLV-Pco and FeLV-945 were distinctive from other strains of FeLV (Figure 5). Of these synapomorphic sites, 2 were in variable region A, which in FeLV-945 defines the specificity required for viral binding to receptors (*25*). Three of the sites were within the proline-rich region, which in FeLV-945 encodes for conformational changes required for FeLV cell entry (*25*). The FeLV-Pco LTR sequences had 1 copy of a 40-bp enhancer element that has been characterized in FeLV-945 (Appendix Figure 2) (*12*). Finally, the exogenous domestic cat FeLV-945 isolate, which FeLV-Pco strains resemble (Figures 4, 5), displays unusual repeat junctions where the transcription factor c-Myb is known to bind in FeLV-945, possibly accelerating the rate of transcription of the virus (Figure 2, Appendix Figure 2) (*12*). FeLV-Pco also contains 1 copy of this repeat junction (Figure 2, panel** A**; Appendix Figure 2), which supports the conclusion that FeLV-Pco is derived from a strain closely related to and perhaps from the pathogenic FeLV-945 domestic cat strain. FeLV-945 is unusual in that its severe pathogenicity does not involve recombination with endogenous FeLV in domestic cats. That FeLV-Pco pathogenesis in pumas is due to a virus similar to FeLV-945 that was not derived from endogenous recombination is consistent with the complete lack of endogenous FeLV sequences in the puma genome.

## Discussion

We genetically characterized the FeLV that emerged in the previously naive free-ranging Florida panther population. According to the retrospective longitudinal antibody and antigen results and the virus’ geographic distribution, the virus was likely introduced into the Florida panther population in 2001 (Figure 1) (*3*). From the earliest detected panthers with FeLV (2001) to the most recent (2005), the FeLV-Pco *env* sequences were nearly identical, which indicates that the source of infection was likely a single domestic cat. FP-96, resident in the Florida Panther National Wildlife Reserve area in January of 2001, was the first panther with exposure detectable by PCR. The virus then spread north and east through the population, affecting individual panthers in Big Cypress (FP-100, FP-119), Seminole Indian Reservation (FP-118), and Okaloacoochee Slough (FP-109, FP-108, FP-115, FP-122, FP-123, FP-132) (Figure 1; Technical Appendix). Texas genetic heritage did not protect infected pumas from disease associated with FeLV; pure Florida panthers and pumas died after having symptoms compatible with FeLV (Figure 1).

Among characterized strains of FeLV, domestic cat FeLV-945 was closest in sequence to FeLV-Pco in the panthers. FeLV-945 in domestic cats was originally isolated as the predominant FeLV species from a geographic cohort of 21 infected domestic cats and is known to cause non–T-cell diseases characterized by degenerative and proliferative changes of myeloid and erythroid origin (*26*). Although FeLV-945 is included among FeLV subgroup A isolates on the basis of cell receptor utilization, its distinctive envelope and LTR sequence signatures differ from those of other FeLV-A strains (*25*). At the amino-terminal of the envelope sequence, the surface glycoprotein, also known as gp70, encodes the receptor-binding domain, within which are 2 variable regions, A and B. These define the specificity required for binding. Further downstream, a proline-rich region encodes for the conformational changes required for viral entry (*25*).

The 10 envelope amino acid residues synapomorphic in FeLV-Pco and FeLV-945 included 2 in variable region A and 3 in the proline-rich region (Figures 2, 5). In FeLV-945 LTR, three 21-bp repeats form 2 junctions: 1 junction is formed by the first repeat and the adjacent second repeat; the other is formed by the second and third repeats. Each junction includes a c-Myb binding site that increases the rate of viral replication through the recruitment of transcriptional coactivator binding protein (cAMP response element) (*11*). FeLV-Pco LTR sequences had 1 copy of the repeat junction (Figure 2, Appendix Figure 2) (*12*). Upstream, LTR transcriptional enhancer elements repeated in tandem have been associated with thymic lymphomas and are found only in 1 copy in non–T-cell disease (*26*). Like FeLV-945, FeLV-Pco lacks this duplication (Figure 2, Appendix Figure 2).

In the panthers, clinical and pathologic findings of FeLV-Pco in this outbreak consisted of FeLV-related diseases of non–T-cell origin. These findings are consistent with the pathologic changes associated with FeLV-945 in the domestic cat. Necropsy findings of FP-115 documented interstitial pneumonia, septicemia, and suppurative lymphadenopathy. Examination of FP-109 1 month before it died found lymphadenopathy, anemia, lymphopenia, and lymphoid hyperplasia. FP-122 had similar findings 1 month before it died, including lymphadenopathy, muscle wasting, and hypercellular bone marrow with >90% hematopoietic cells. FP-132 necropsy findings included severe pallor (indicative of anemia), bronchointerstitial pneumonia, abscesses, lymphadenopathy, and hypercellular bone marrow with >90% hematopoietic cells (*5*). FeLV-Pco is therefore similar to the unique and virulent domestic cat strain FeLV-945 of FeLV subgroup A, in *env* and *LTR* sequence and in non–T-cell disease outcome. In the domestic cat, FeLV-945 causes multicentric lymphoma, myeloproliferative disorder, and anemia and has never been associated with thymic lymphoma (*26*). These findings shared between FeLV-945 and FeLV-Pco implicate the 10 identified amino acid synapomorphies (Figure 5) as plausible determinants of disease. Further study of these *env* regions from T-cell and non–T-cell disease manifestations of FeLV occurring in comparative felid species is warranted and may elucidate the key sequence determinants of disease outcome in FeLV.

The role of FIV-related immune suppression, if any, in this outbreak is uncertain. Although recent studies of T-lymphocyte profiles in FIV-infected wild lions and pumas suggest that CD4 depletion occurs (*27*), our survey found that co-infection with FIV was present in 2 but absent in 3 FeLV-associated deaths. FIV-positive panthers could have served as a reservoir for the spread of FeLV through the population because the earliest detected FeLV-exposed panthers (FP-96 and FP-99) were FIV positive. Furthermore, the first panther (FP-115) detected with FeLV-compatible disease in the Okaloacoochee Slough State Forest region was positive for FIV and FeLV for at least 6 months.

An FIV serosurvey suggested an overall increase in the prevalence of FIV in Florida panthers in recent years. During 1999–2000, 3 (15%) of 20 panthers tested had FIV-positive results by Western blot. In contrast, 13 (76%) of 17 panthers tested during 2004–2005 in the FeLV-endemic Okaloacoochee Slough State Forest region (Figure 1) were FIV positive (*5*). These results could support a role for FIV-mediated immune depletion in FeLV pathogenesis. In domestic cats, FIV and FeLV co-infections have resulted in conflicting interpretations (*28*–*32*). In contrast to FIV, which is found in many species of wild felids (*33*), FeLV in nondomestic felids has been reported only a few times, in captive cats, with documented or suspected exposure to infected domestic cats (*5*). Serologic survey of free-ranging populations found an absence of FeLV in pumas in California (*34*), among felids in Botswana (*35*), and among 38 free-ranging Florida panthers sampled during 1978–1991 (*3*). However, Jessup et al. (*36*) document a case of FeLV in a young adult male free-ranging puma captured from a college campus in Sacramento, California. Necropsy of this cougar found generalized lymphadenopathy and lymphoproliferative disease. These necropsy results are consistent with and similar to the clinical findings of the FeLV-positive panthers reported here.

The outbreak of FeLV in the previously naive population of endangered Florida panthers raised questions about management of free-ranging pumas. In response, the Florida Department of Fisheries and Wildlife began a widespread vaccination program of Florida panthers; no additional FeLV cases have since been detected among them (*5*).

This emerging disease outbreak was characterized by 2 factors. First, because of its unique heritage and popularity, the Florida panther has been the most intensively monitored wild felid in North America. Second, the extensive veterinary surveillance of the domestic cat has provided powerful models for studying infectious diseases relevant to understanding human health and disease, including retroviruses such as FeLV (*37*). Although future cross-species transmission events among wild and domestic carnivore populations may be unavoidable, our understanding of pathogen and host genetic determinants may also be greatly enhanced by the recent release of the genome sequence of the domestic cat (*38*). Combining progress in biomedical genomics with intensive studies of wild species can provide insights into emerging pathogens that affect wild, domestic, and human hosts.

## Supplementary Material

Technical Appendix Proviral PCR screening, 61 puma samples, 1988–2006*

Appendix Figure 1 Alignment of all PCR-positive puma envelope (env) sequence with domestic cat feline leukemia virus (FeLV)A-945 = AY374189, FeLVA-3281 = M18248. FCA-IXODES was a FeLV-positive domestic cat from Florida.

Appendix Figure 2 Top) Alignment of 1,794 bp of env nucleotide sequences corresponding to feline leukemia virus (FeLVA)-945 (AY662447) index sequence 154-1,869 bp. The shaded areas identify regions (indels) where panther FeLV (FeLV-Pco) sequences resemble those of FeLV-A, which rules out recombination with dissimilar endogenous FeLV sequences as represented in enFeLV-AGTT. Bottom) Panther sequences, with year of sampling (for example, FeLV-Pco-1058-03 was sampled in 2003); domestic cat subgroup A (FeLVA-945 and FeLVA-61E), recombinant (FeLVB-GA), and endogenous (enFeLV-AGTT) sequences are also shown. Matches to the reference sequence (Pco-1058-02) are indicated by a dot. Gaps are indicated by a dash.

Expanded Figure
