# Supplementary material for: Genetic Characterization of Feline Leukemia Virus from Florida Panthers
Source: Emerg Infect Dis. 2008 Feb;14(2):252–9. doi: 10.3201/eid1402.070981 (PMC2600209; doi:10.3201/eid1402.070981)
Supplement: Technical Appendix — Proviral PCR screening, 61 puma samples, 1988–2006* [file 07-0981_Techapp-s1.pdf]

Technical Appendix

Table. Proviral PCR screening, 61 puma samples, 1988–2006*

FP ID†	LGD ID‡	Date§	Range¶	Source#	PCR**	Antigen**††	Antibody titer**
FP25	Pco-116	2/16/88	F	WBC	Neg	ND	ND
FP18	Pco-68	1/23/89	F	BY	Neg	ND	ND
FP28	Pco-154	3/11/89	S	BY	Neg	ND	ND
FP21	Pco-75	5/23/89	E	CT	Neg	ND	ND
FP29	Pco-155	1/10/91	S	BY	Neg	ND	ND
	Pco-409	8/17/91	Peru	CT	Neg	ND	ND
FP54	Pco-487	4/7/92	F	BY	Neg	ND	ND
FP12	Pco-20	1/4/93	F	BY	Neg	ND	ND
FP31	Pco-157	1/6/93	F	BY	Neg	ND	ND
	Pco-554	1/23/93	Nicaragua	WBC	Neg	ND	ND
	Pco-579	5/15/93	Argentina	WBC	Neg	ND	ND
	Pco-581	5/19/93	Paraguay	WBC	Neg	ND	ND
	Pco-582	5/19/93	Uruguay	WBC	Neg	ND	ND
TX33	Pco-741	1/4/96	Texas	WBC	Neg	ND	ND
WC-0	Pco-742	4/14/97	S	BY	Neg	ND	ND
FP78	Pco-908	2/16/99	F	BY	Neg	ND	ND
WC-06	Pco-926	7/19/99	S	BY	Neg	ND	ND
WC-03	Pco-923	1/13/00	S	BY	Neg	ND	ND
FP61	Pco-898	2/17/00	E	BY	Neg	ND	ND
FP89	Pco-969	3/2/00	BC-S	BY	Neg	ND	ND
FP92	Pco-916	4/6/00	F	BY	Neg	ND	ND
TX107	Pco-736	4/19/00	F	BY	Neg	ND	ND
TX105	Pco-739	12/1/00	E	BY	Neg	ND	ND
FP96	Pco-972	1/8/01	F	BY	Pos	Neg	0.337
FP99	Pco-990	1/27/01	F	BY	Neg	ND	0.296
FP100	Pco-991	2/1/01	BC-S	BY	Pos	Neg	0.3
FP101	Pco-992	2/5/01	BC-S	BY	Neg	ND	ND
FP102	Pco-996	2/20/01	BC-S	BY	Neg	ND	ND
FP104	Pco-1000	4/3/01	BC-S	BY	Neg	Neg	0.292
UCFP39	Pco-1004	5/7/01	BC-S	LN	Neg	Neg	ND
FP107	Pco-971	11/1/01	F	BY	Neg	Neg	0.324
FP96	Pco-972	11/3/01	F	BY	Pos	Neg	ND
FP108	Pco-994	11/6/01	BC-S	BY	Neg	Neg	0.273
FP78	Pco-908	12/14/01	F	BY	Neg	Neg	0.454
FP96	Pco-972	1/18/02	F	SP	Pos	Neg	ND
FP110	Pco-984	2/13/02	O	BY	Neg	Neg	ND
FP111	Pco-1023	2/14/02	O	nvPBL	Neg	Neg	0.224
FP112	Pco-1024	2/25/02	BC-S	BY	Neg	Neg	ND
K109FP73	Pco-1025	3/3/02	BC-S	BY	Neg	Neg	ND
UCFP46	Pco-1029	4/10/02	BC-S	SP	Neg	ND	ND
K12FP78	Pco-1038	10/23/02	BC-S	BY	Neg	ND	0.242
FP110	Pco-984	11/25/02	O	BY	Pos	Neg	ND
FP115	Pco-1058	11/26/02	O	BY	Pos	Pos	0.499
FP82	Pco-962	12/6/02	O	BY	Neg	ND	0.262
TX106	Pco-733	1/9/03	F	BY	Neg	ND	ND
FP109	Pco-1022	1/24/03	O	BM	Pos	Pos	0.546
FP118	Pco-1060	3/6/03	S	BY	Pos	Neg	0.157
FP119	Pco-1064	4/4/03	BC-N	BY	Pos	Neg	0.125
FP115	Pco-1058	5/27/03	O	BM	Pos	Pos	ND
FP118	Pco-1060	5/27/03	S	SP	Pos	Neg	ND
K151	Pco-1073	6/12/03	O	BY	Neg	ND	ND

UCFP57	Pco-1076	6/17/03	BC-N	LN	Neg	ND	ND
UCFP58	Pco-1084	6/30/03	S	SP	Neg	ND	ND
FP121	Pco-1085	12/2/03	S	BY	Neg	ND	ND
FP117	Pco-1059	12/3/03	BC-N	BY	Neg	Neg	0.17
FP100	Pco-991	1/6/04	BC-N	BY	Pos	Neg	ND
FP122	Pco-1087	2/2/04	O	BY	Pos	Pos	ND
FP123	Pco-1088	2/2/04	O	BY	Pos	Pos	ND
FP124	Pco-1091	2/13/04	B	BY	Neg	ND	ND
FP127	Pco-1094	2/16/04	B	BY	Neg	ND	ND
FP71	Pco-1095	2/17/04	S	BY	Neg	ND	ND
FP131	Pco-1097	3/10/04	F	BY	Neg	ND	ND
FP132	Pco-1098	3/18/04	O	BY	Pos	Neg	ND
FP78FP83	Pco-914	3/31/04	BC-S	BY	Neg	ND	ND
UCFP65	Pco-1103	4/6/04	F	SP	Neg	ND	ND
FP113	Pco-1037	4/7/04	BC-N	BY	Neg	ND	ND
FP117	Pco-1059	7/29/04	BC-N	BM	Neg	ND	ND
FP132	Pco-1098	8/1/04	O	SP	Pos	Pos	ND
FP119	Pco-1064	11/17/04	BC-N	BY	Neg	Neg	ND
UCFP43	Pco-1016	8/30/05	P	BY	Neg	Neg	0.277
FP67	Pco-722	4/23/06	P	SP	Neg	Neg	0.26

*The 376-bp amplification of *env* was confirmed by sequencing.
†FP ID, Florida panther identification.
‡LGD ID, Laboratory of Genomic Diversity identification.
§Date, month/day/year format.
¶Range, E, Everglades National Park; F, Florida Panther National Wildlife Refuge; O, Okaloacoochee Slough State Forest; P, private lands; S, Big Cypress Seminole Indian Reservation; BC-N, Big Cypress North, BC-S, Big Cypress South.
#Source of DNA isolation: WBC, white blood cell; BY, buffy coat; CT, clot; BM, bone marrow; SP, spleen; LN, lymph node; nvPBMCs, nonviable peripheral blood mononuclear cells.
**Neg, negative; Pos, positive.
††ND, not detemined. Antibody titer >0.25 was considered positive (*1*).

Reference

1. Cunningham MW, Brown MA, Shindle DB, Terrell SP, Hayes KA, Ferree BC, et al. Epizootiology and management of feline leukemia virus in the Florida puma. J Wildl Dis. In press.
