# Supplementary figures and images for: Genetic Characterization of Feline Leukemia Virus from Florida Panthers
Source: Emerg Infect Dis. 2008 Feb;14(2):252–9. doi: 10.3201/eid1402.070981 (PMC2600209; doi:10.3201/eid1402.070981)

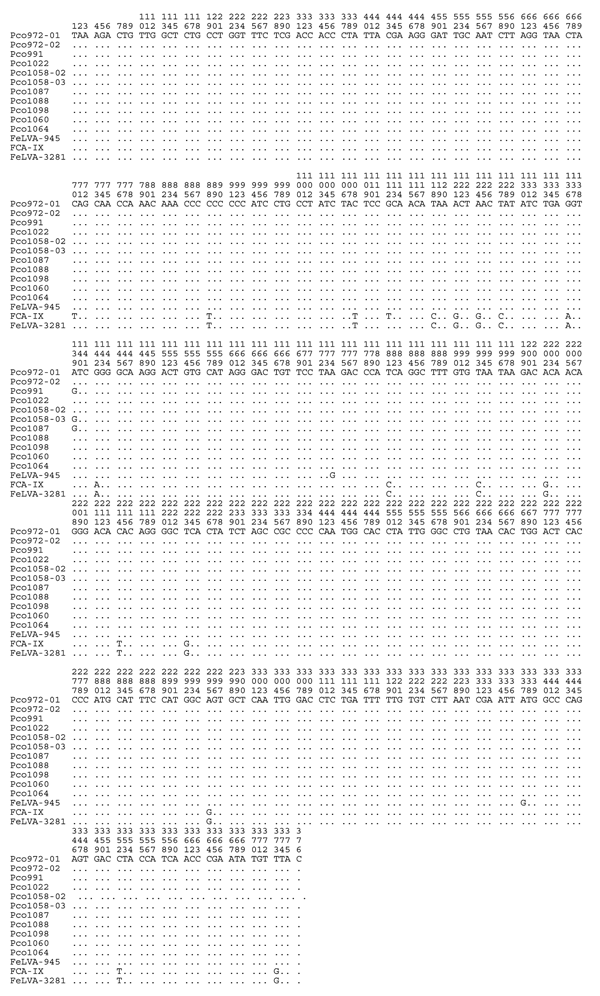

Supplement: Appendix Figure 1 — Alignment of all PCR-positive puma envelope (env) sequence with domestic cat feline leukemia virus (FeLV)A-945 = AY374189, FeLVA-3281 = M18248. FCA-IXODES was a FeLV-positive domestic cat from Florida. [file 07-0981_app1-s2.gif]

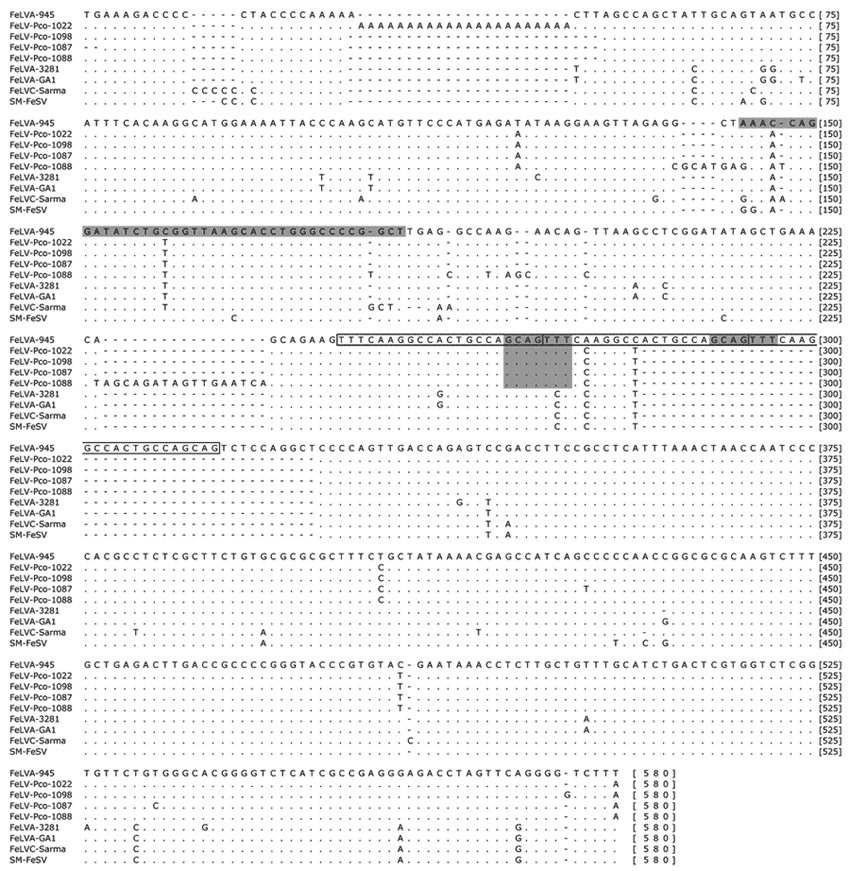

Supplement: Appendix Figure 2 — Top) Alignment of 1,794 bp of env nucleotide sequences corresponding to feline leukemia virus (FeLVA)-945 (AY662447) index sequence 154-1,869 bp. The shaded areas identify regions (indels) where panther FeLV (FeLV-Pco) sequences resemble those of FeLV-A, which rules out recombination with dissimilar endogenous FeLV sequences as represented in enFeLV-AGTT. Bottom) Panther sequences, with year of sampling (for example, FeLV-Pco-1058-03 was sampled in 2003); domestic cat subgroup A (FeLVA-945 and FeLVA-61E), recombinant (FeLVB-GA), and endogenous (enFeLV-AGTT) sequences are also shown. Matches to the reference sequence (Pco-1058-02) are indicated by a dot. Gaps are indicated by a dash. [file 07-0981_app2-s3.gif]

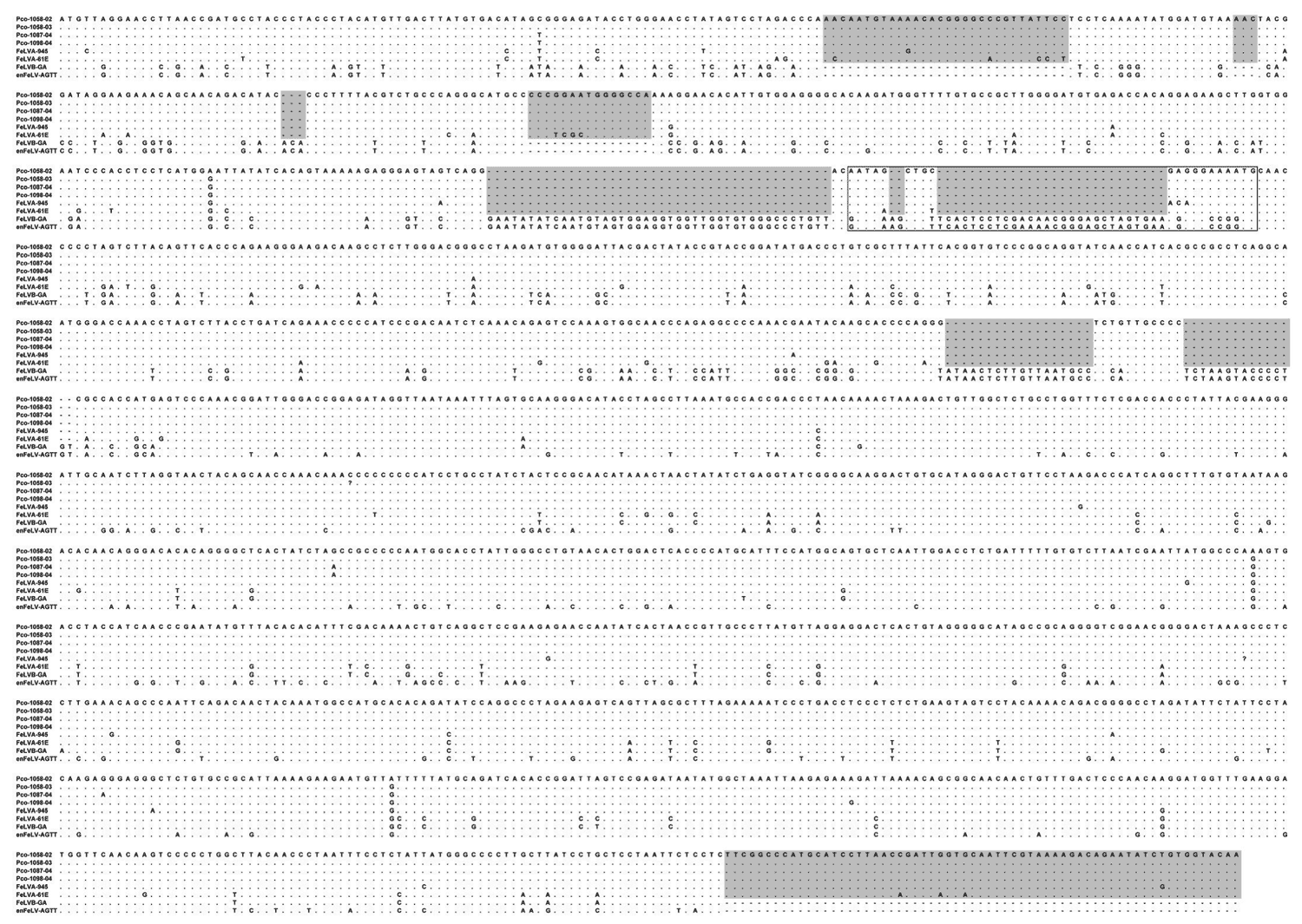

Supplement: Expanded Figure [file 07-0981_app3-s4.gif]
